# Supplementary material for: Functional dissection of the conserved C. elegans LEM-3/ANKLE1 nuclease reveals a crucial requirement for the LEM-like and GIY-YIG domains for DNA bridge processing
Source: Nucleic Acids Res. 2025 Apr 7;53(6):gkaf265. doi: 10.1093/nar/gkaf265 (PMC11975286; doi:10.1093/nar/gkaf265)
Supplement: gkaf265_Supplemental_Files [file gkaf265_supplemental_files.zip › Song et al - suppl data 2025-03-20.pdf]

Functional dissection of the conserved *C. elegans* LEM-3/ANKLE1 nuclease reveals a crucial requirement for the LEM-like and GIY-YIG domains for DNA bridge processing.

Junfang Song[1,*,#], Peter Geary[2,3*], Khadisha Salemova[2,4], John Rouse[1], Ye Hong[5], Stéphane G.M. Rolland [2,6] and Anton Garter [2,3,6,**]**

Supplementary data:

Figure S1

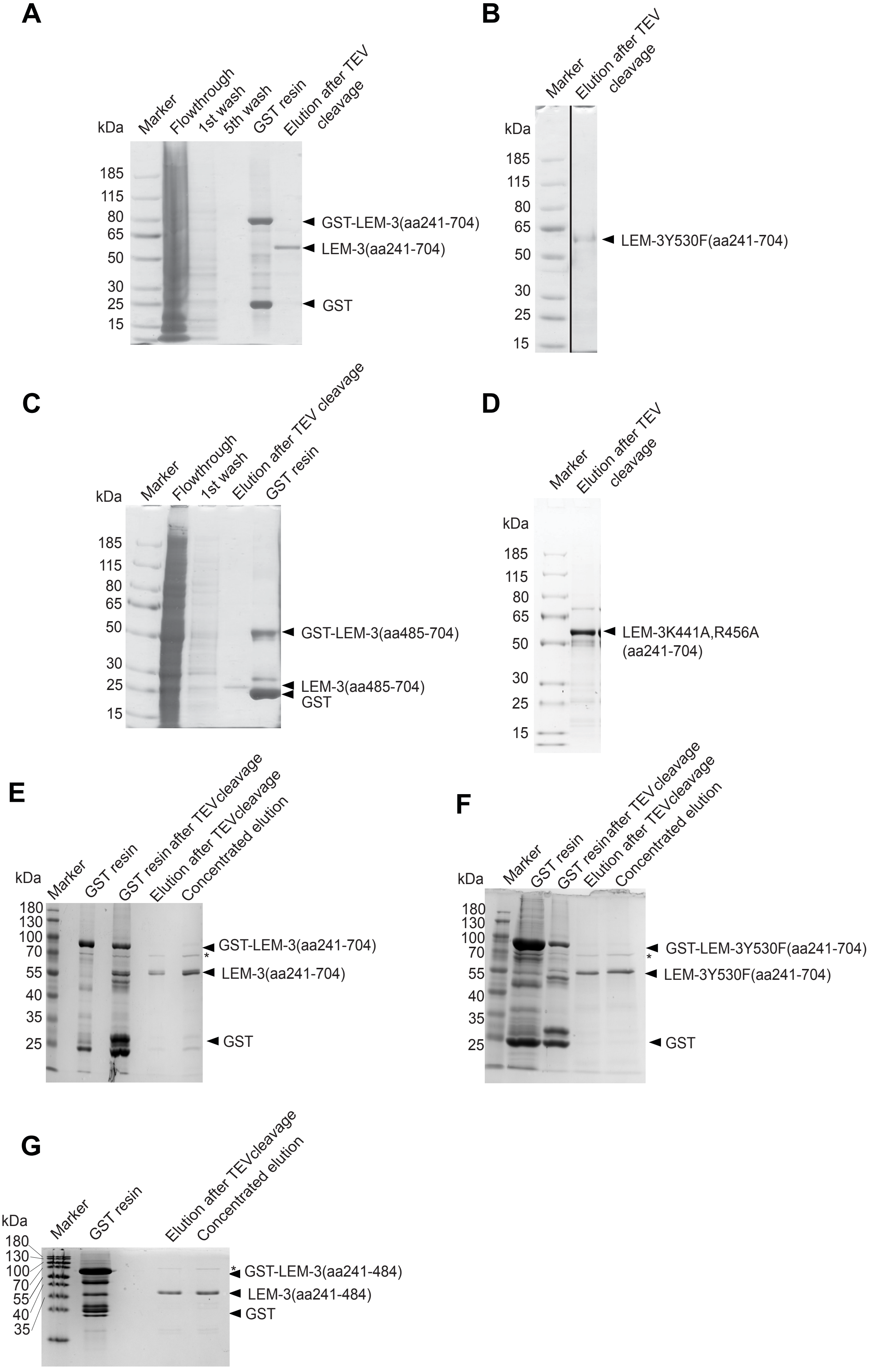
A
Marker
Flowthrough
1st wash
5th wash
GST resin
Elution after TEV cleavage
kDa
185
115
80
65
50
30
25
15
GST-LEM-3(aa241-704)
LEM-3(aa241-704)
GST
B
Marker
Elution after TEV cleavage
kDa
185
115
80
65
50
30
25
15
LEM-3Y530F(aa241-704)
C
Marker
Flowthrough
1st wash
Elution after TEV cleavage
GST resin
kDa
185
115
80
65
50
30
25
15
GST-LEM-3(aa485-704)
LEM-3(aa485-704)
GST
D
Marker
Elution after TEV cleavage
kDa
185
115
80
65
50
30
25
15
LEM-3K441A,R456A (aa241-704)
E
Marker
GST resin
GST resin after TEV cleavage
Elution after TEV cleavage
Concentrated elution
kDa
180
130
100
70
55
40
35
25
GST-LEM-3(aa241-704)
*
LEM-3(aa241-704)
GST
F
Marker
GST resin
GST resin after TEV cleavage
Elution after TEV cleavage
Concentrated elution
kDa
180
130
100
70
55
40
35
25
GST-LEM-3Y530F(aa241-704)
*
LEM-3Y530F(aa241-704)
GST
G
Marker
GST resin
Elution after TEV cleavage
Concentrated elution
kDa
180
130
100
70
55
40
35
*
GST-LEM-3(aa241-484)
LEM-3(aa241-484)
GST

Figure S1: Purification of the different LEM-3 derivatives used in this study using the baculovirus-based expression system (related to Figures 1, 3, ,4 and 5). Flowthrough, 1st wash, 5th wash, GST resin, and Elution after TEV cleavage are indicated **(A)** LEM-3(241-704), **(B)** LEM-3Y530F(241-704), **(C)** LEM-3(485-704), **(D)** LEM-3K441A; R456A(241-704), **(E)** newly purified LEM-3(241-704), **(F)** newly purified LEM-3Y530F(241-704), **(G)** LEM-3(241-484). Uncropped versions of panels B and D are shown in Supplementary Figures S11A and B, respectively.

Figure S2

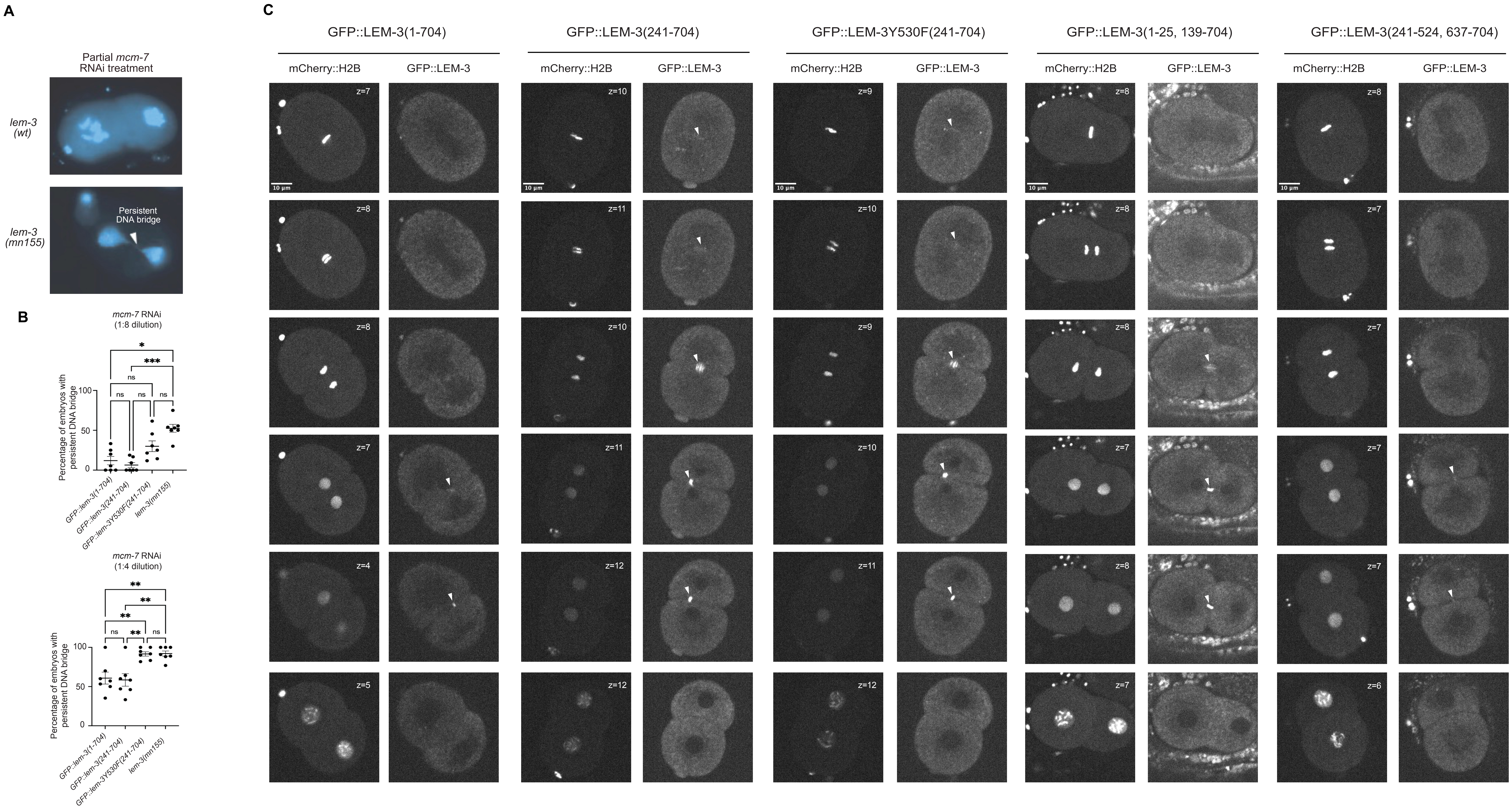
A
Partial *mcm-7* RNAi treatment
lem-3 (wt)
lem-3 (mn155)
Persistent DNA bridge
B
mcm-7 RNAi (1:8 dilution)
mcm-7 RNAi (1:4 dilution)
Percentage of embryos with persistent DNA bridge
*
***
**
ns
0
50
100
GFP::lem-3(1-704)
GFP::lem-3(241-704)
GFP::lem-3Y530F(241-704)
lem-3(mn155)
C
GFP::LEM-3(1-704)
GFP::LEM-3(241-704)
GFP::LEM-3Y530F(241-704)
GFP::LEM-3(1-25, 139-704)
GFP::LEM-3(241-524, 637-704)
mCherry::H2B
GFP::LEM-3
10 µm

Figure S2: Partial *mcm-7* RNAi experiment and time-lapse imaging of the first cell division of various GFP::LEM-3 mutant strains in the absence of DNA bridges (related to Figure 1). (A) Upon partial *mcm-7* RNAi, persistent DNA bridges are revealed by DAPI staining. **(B)** Analysis of the sensitivity to partial *mcm-7* RNAi of the indicated LEM-3 derivatives. Different dilutions of RNAi were used (*mcm-7* RNAi was diluted 1:4 and 1:8 with *mock* RNAi), and the percentage of 2-cell stage embryos with persistent DNA bridges was determined (n=7 experiments (in each experiment, ~10-15 embryos were analyzed), mean and SEM are shown. For 1:4 dilution, ns = not significant and ** = $p < 0.01$ by one-way ANOVA with Tukey's multiple comparisons test. For 1:8 dilution, ns = not significant and * $p < 0.05$ and *** $p < 0.001$ by Kruskal-Wallis with Dunn's multiple comparison). **(C)** mCherry:H2B and GFP channels are shown. The time (post-anaphase onset) and the Z-position in the Z-stack for each snapshot are indicated. For all the GFP::LEM-3 mutant strains and the wild-type GFP::LEM-3 strain, the LUT (95-150) was used.

Figure S3

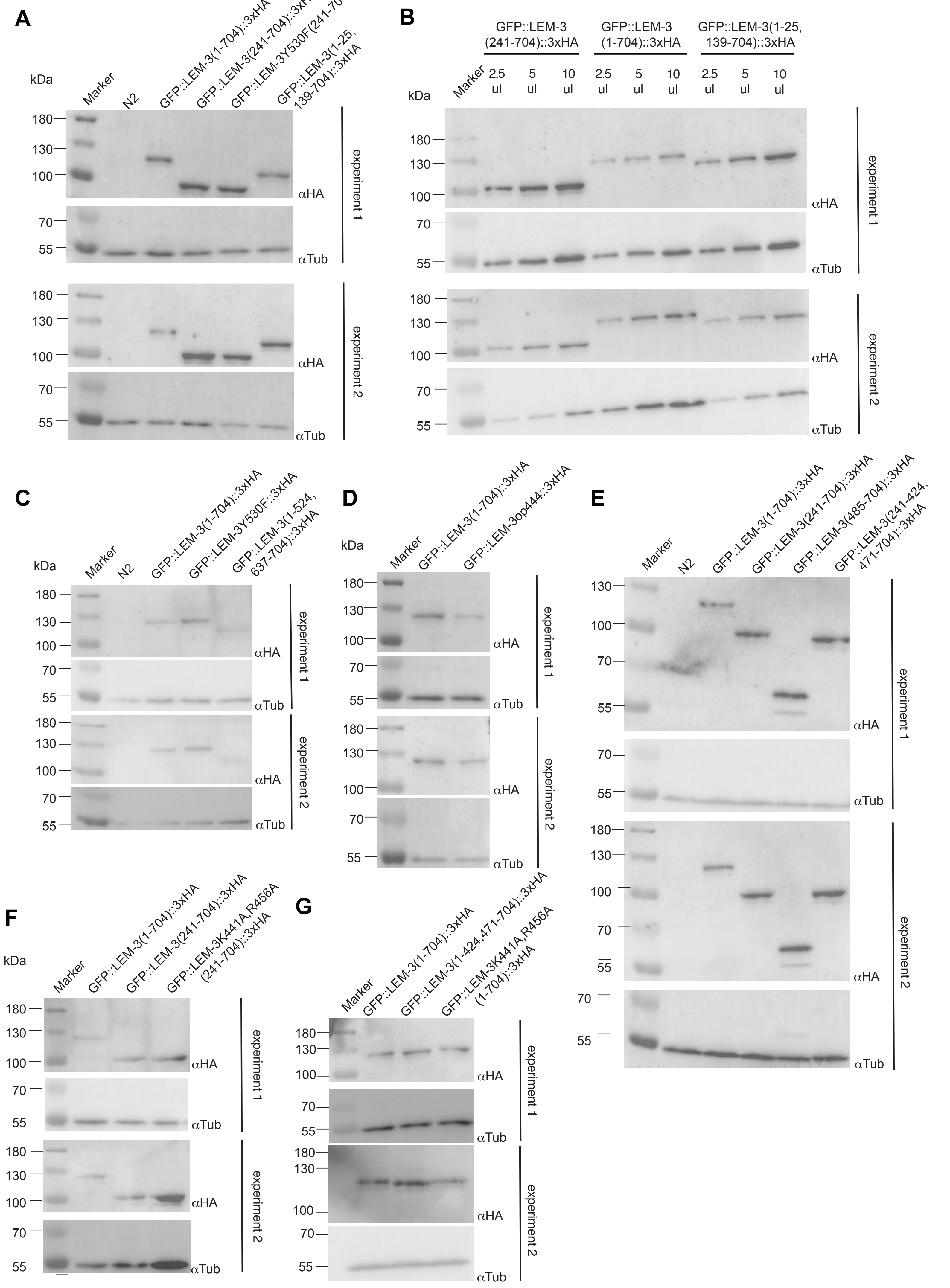
A
kDa
Marker
N2
GFP::LEM-3(1-704)::3xHA
GFP::LEM-3(241-704)::3xHA
GFP::LEM-3Y530F(241-704)::3xHA
GFP::LEM-3(1-25, 139-704)::3xHA
180
130
100
70
55
αHA
αTub
experiment 1
experiment 2
B
GFP::LEM-3 (241-704)::3xHA
GFP::LEM-3 (1-704)::3xHA
GFP::LEM-3(1-25, 139-704)::3xHA
2.5 ul
5 ul
10 ul
C
GFP::LEM-3(1-704)::3xHA
GFP::LEM-3Y530F::3xHA
GFP::LEM-3(1-524, 637-704)::3xHA
D
GFP::LEM-3(1-704)::3xHA
GFP::LEM-3op444::3xHA
E
GFP::LEM-3(1-704)::3xHA
GFP::LEM-3(241-704)::3xHA
GFP::LEM-3(485-704)::3xHA
GFP::LEM-3(241-424, 471-704)::3xHA
F
GFP::LEM-3(1-704)::3xHA
GFP::LEM-3(241-704)::3xHA
GFP::LEM-3K441A,R456A (241-704)::3xHA
G
GFP::LEM-3(1-704)::3xHA
GFP::LEM-3(1-424,471-704)::3xHA
GFP::LEM-3K441A,R456A (1-704)::3xHA

Figure S3: Western-blot analysis of the level of the different GFP::LEM-3 derivatives (Related to Figures 1, 2, 4, 5, and 6). (A) Analysis of the level of GFP::LEM-3(1-704)::3xHA, GFP::LEM-3(241-704)::3xHA, GFP::LEM-3Y530F(241-704)::3xHA and GFP::LEM-3(1-25,139-704)::3xHA derivatives. **(B)** Analysis of the level of GFP::LEM-3(1-704)::3xHA, GFP::LEM-3(241-704)::3xHA and GFP::LEM-3(1-25,139-704)::3xHA derivative. Different volumes (2.5ul, 5ul and 10ul) of the samples were loaded on the gel. **(C)** Analysis of the level of GFP::LEM-3(1-704)::3xHA, GFP::LEM-3Y530F::3xHA and GFP::LEM-3(1-524,637-704)::3xHA derivatives. **(D)** Analysis of the level of GFP::LEM-3(1-704)::3xHA and GFP::LEM-3op444::3xHA derivatives. **(E)** Analysis of the level of GFP::LEM-3(1-704)::3xHA, GFP::LEM-3(241-704)::3xHA, GFP::LEM-3(485-704)::3xHA and GFP::LEM-3(241-424,471-704)::3xHA derivatives. **(F)** Analysis of the level of GFP::LEM-3(1-704)::3xHA, GFP::LEM-3(241-704)::3xHA and GFP::LEM-3K441A,R456A(241-704)::3xHA derivatives. **(G)** Analysis of the level of GFP::LEM-3(1-704)::3xHA, GFP::LEM-3(1-424,471-704)::3xHA and GFP::LEM-3K441A,R456A(1-704)::3xHA derivatives. For all panels, as described in the methods, the different strains were grown at 25°C for 3 days. Total proteins were extracted and analyzed by SDS-PAGE and Western using anti-HA and anti-Tubulin antibodies. For all panels, two independent experiments were performed. (N2 corresponds to the non-transgenic wildtype strain).

Figure S4

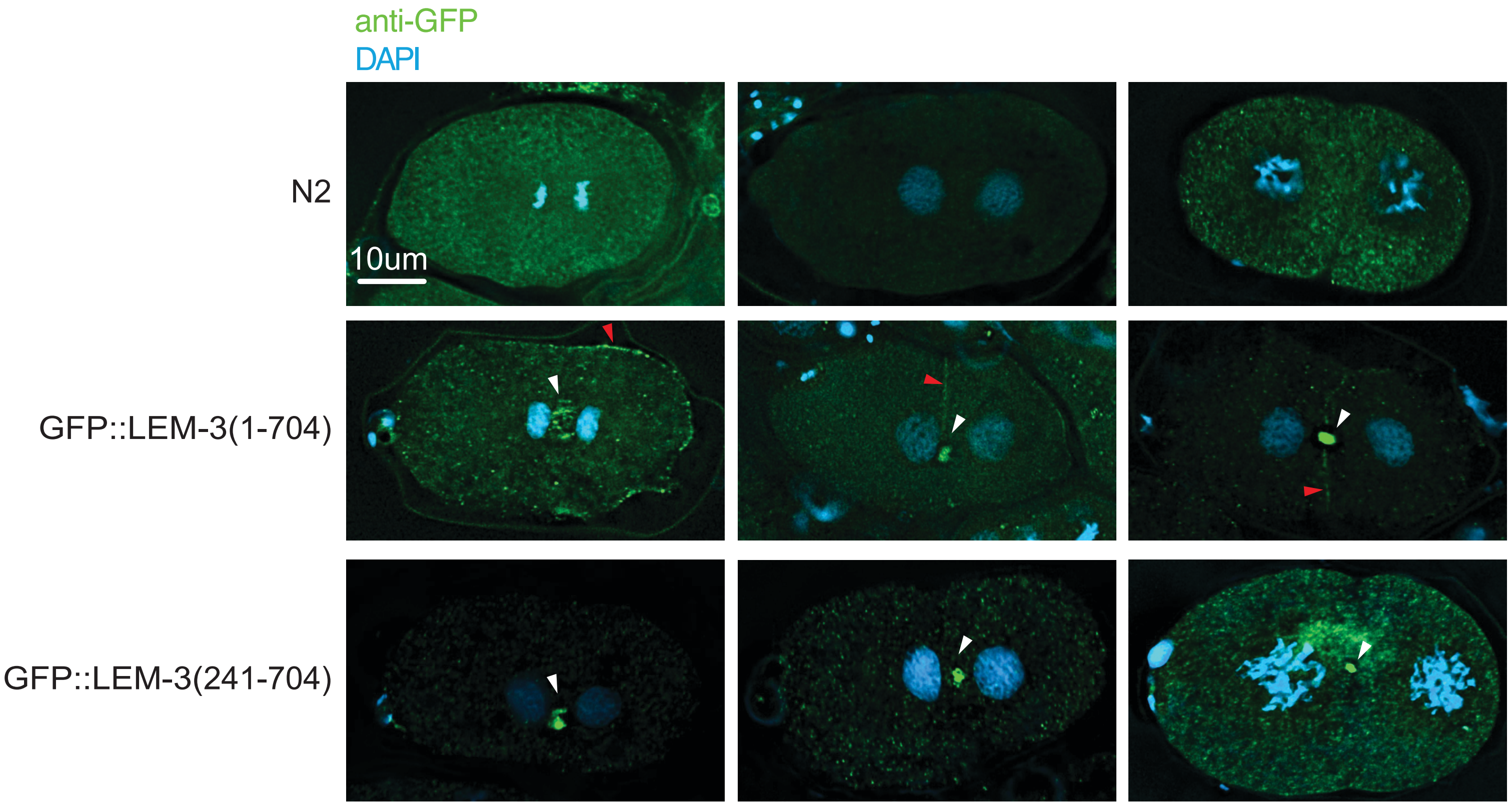

Figure S4: Immunostaining of GFP::LEM-3(1-704) and GFP::LEM-3(241-704) (related to Figure 1). As described in the methods, embryos were subjected to the ‘freeze-crack’ method and stained with anti-GFP antibodies. Early stages of the first cell division were imaged by epifluorescence. Deconvolved images are shown. N2 embryos were used as a control. White arrowheads indicate the LEM-3 foci at the midzone and midbody, while red arrowheads indicate LEM-3 at the plasma membrane.

Figure S5

A

```
                Y530                 Y556 K559              R564 H568
LEM-3     525  YNAFCYLIMDP 535   553 SIFYVGKGSK 562    561 SKNRPLAHFIDARNERRD 578
ANKLE1    449  KSSFTYLLLDP 459   484 AIFYVGKGTR 492    491 TRARPYVHLWEALGHHG 507
Cg-SLX1     9  DFYGCYLLQSI 19     23 QSFYIGSTPN 32      32 NPVRRLRQHNGS 44
T4endoII  32   KYNVIYAIAIN 42     44 ELVYIGKTKN 53      54  LRKRINYYRTAI 65
UvrC       14  EEPGVYIFKNK 24     26 VPIYIGKAKR 35      35 RLSNRLRSYLNP 46
Hpy188I   58   NEGLVYVFVIQ 68     70 KIFKIGHSIT 79      81  ITKRVQSYNCG 91
                 β sheet1            β sheet2               α helix1

                                            E620
LEM-3    602 IPRHEISHGV   611   613 DEEAFVREASIIEAVKLKNLRNKKAGEFH 641
ANKLE1   534 VSLHCFQHVV   543   539 FQHVVAVEAYTREACIVEALGIQTLTNQK 567
Cg-SLX1   60 WEMVAIVYGF   69    71  SRIAALQFEHAWQHGYQTRYIKSQDRVVKT 100
T4endoII  90 VEFYARQCFN   99    112  ATIDLEEPLFIKLFNPPWNI 131
UvrC      59 DELETIVVMN   68    68  NREAFILEANLIKKYRPKYNV 89
Hpy188I  119 VQVYAFFPEQ   128   143  STSKRAENVILENFIKNHNKKPIGCT 168
              β sheet3                  α helix2
```

B ANKLE1 GIY-YIG domain

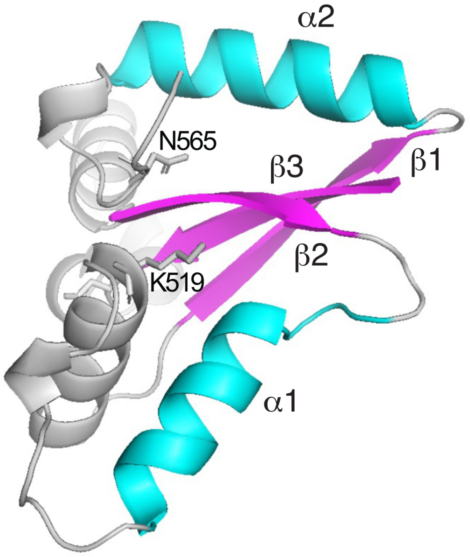

C LEM-3Y530F(485-704)

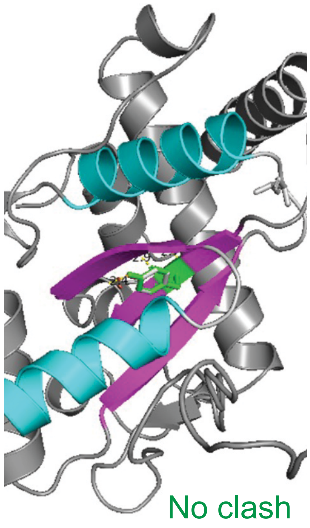

B LEM-3Y556F(485-704)

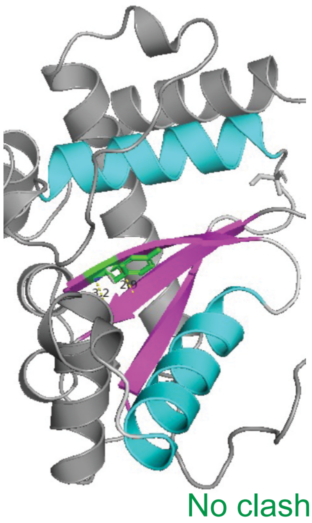

E LEM-3G558A(485-704)

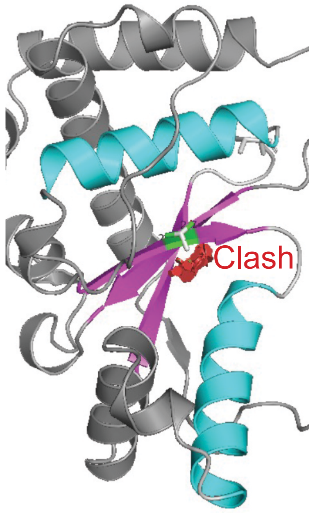

F LEM-3L659F(485-704)

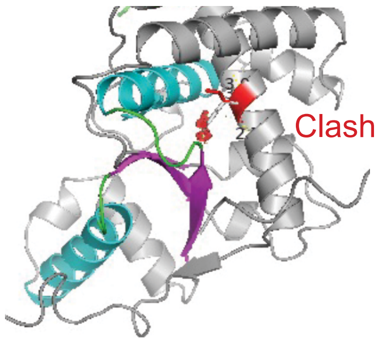

Figure S5: Conservation of the LEM-3 GIY-YIG domain and predicted structure of various LEM-3 mutants (related to Figure 2). (A) Sequence alignment of the GIY-YIG domain of different GIY-YIG nucleases **(B)** Predicted structure by AlphaFold of ANKLE1 GIY-YIG domain with the position of the K519 and N565 residues. **(C-F)** Predicted structure by AlphaFold of LEM-3Y530F(485-704), LEM-3Y556F(485-704), LEM-3G558A(485-704) and LEM-3L659F(485-704). Predicted structural clashes are indicated.

Figure S6

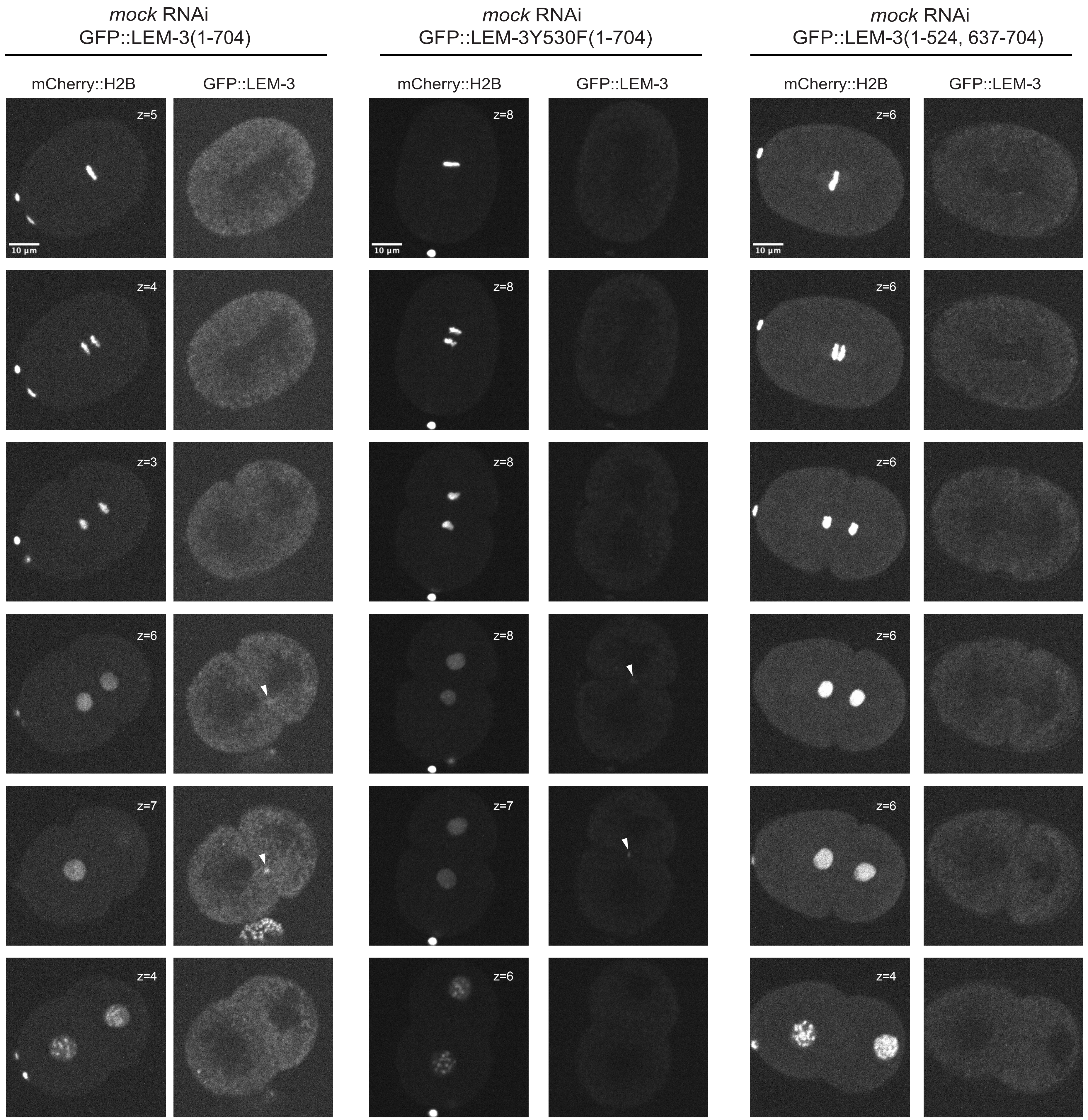
mock RNAi
GFP::LEM-3(1-704)
mock RNAi
GFP::LEM-3Y530F(1-704)
mock RNAi
GFP::LEM-3(1-524, 637-704)
mCherry::H2B
GFP::LEM-3
mCherry::H2B
GFP::LEM-3
mCherry::H2B
GFP::LEM-3
z=5
z=4
z=3
z=6
z=7
z=4
z=8
z=8
z=8
z=8
z=7
z=6
z=6
z=6
z=6
z=6
z=6
z=4
10 µm
10 µm
10 µm

Figure S6: Time-lapse imaging of the first cell division of various GFP::LEM-3 mutant strains in the absence of DNA bridges (related to Figure 2). mCherry:H2B and GFP channels are shown. The time (post-anaphase onset) and the Z-position in the Z-stack for each snapshot are indicated. For all the GFP::LEM-3 mutant strains and the wild-type GFP::LEM-3 strain, the LUT (95-150) was used.

Figure S7

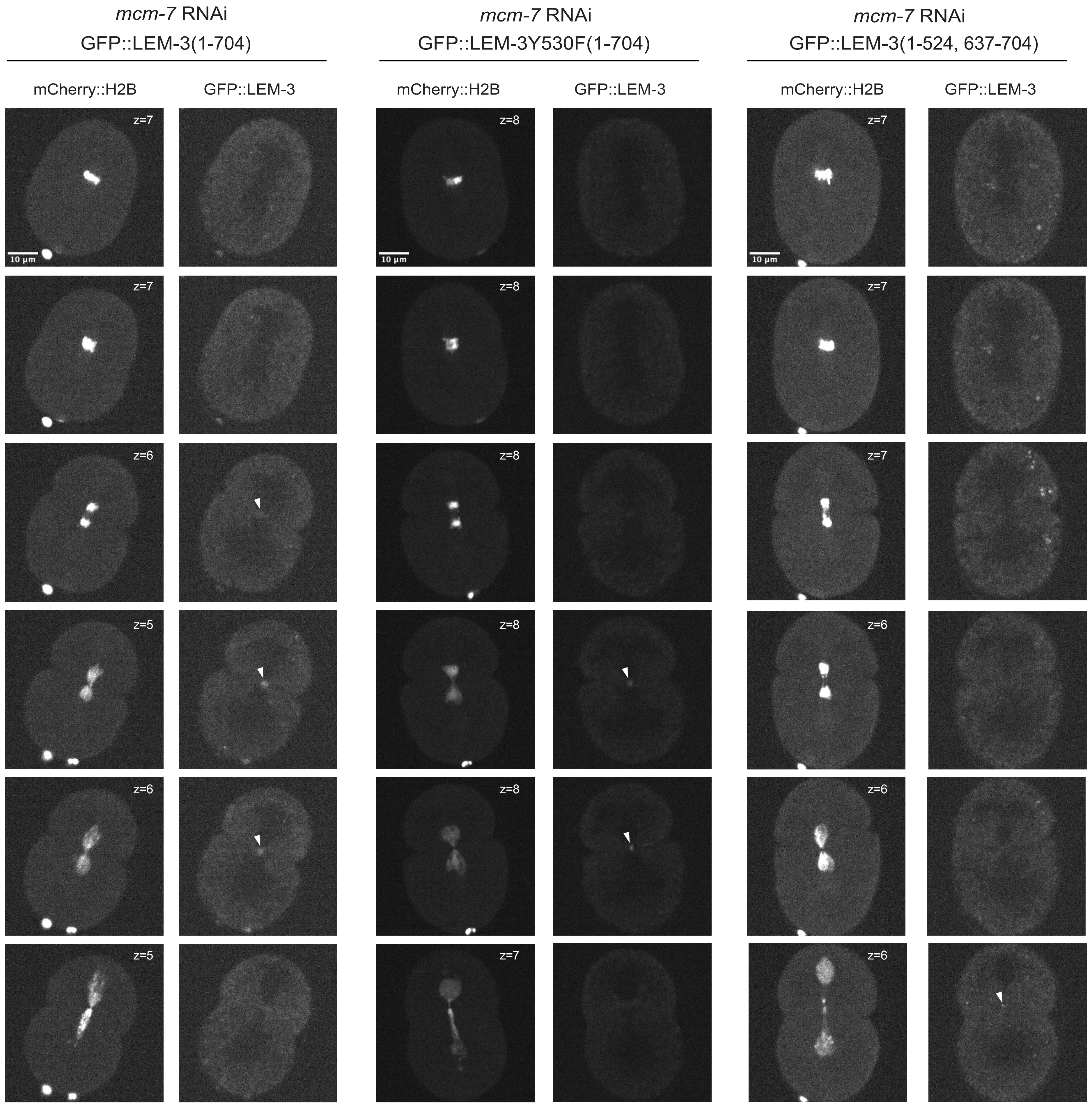

mcm-7 RNAi
GFP::LEM-3(1-704)
mcm-7 RNAi
GFP::LEM-3Y530F(1-704)
mcm-7 RNAi
GFP::LEM-3(1-524, 637-704)
mCherry::H2B
GFP::LEM-3
mCherry::H2B
GFP::LEM-3
mCherry::H2B
GFP::LEM-3
z=7
z=8
z=7
10 µm
z=7
z=8
z=7
z=6
z=8
z=7
z=5
z=8
z=6
z=6
z=8
z=6
z=5
z=7
z=6

Figure S7: Time-lapse imaging of the first cell division of various GFP::LEM-3 mutant strains in the presence of DNA bridges (related to Figure 2). mCherry:H2B and GFP channels are shown. The time (post-anaphase onset) and the Z-position in the Z-stack for each snapshot are indicated. For all the GFP::LEM-3 mutant strains and the wild-type GFP::LEM-3 strain, the LUT (95-150) was used.

Figure S8

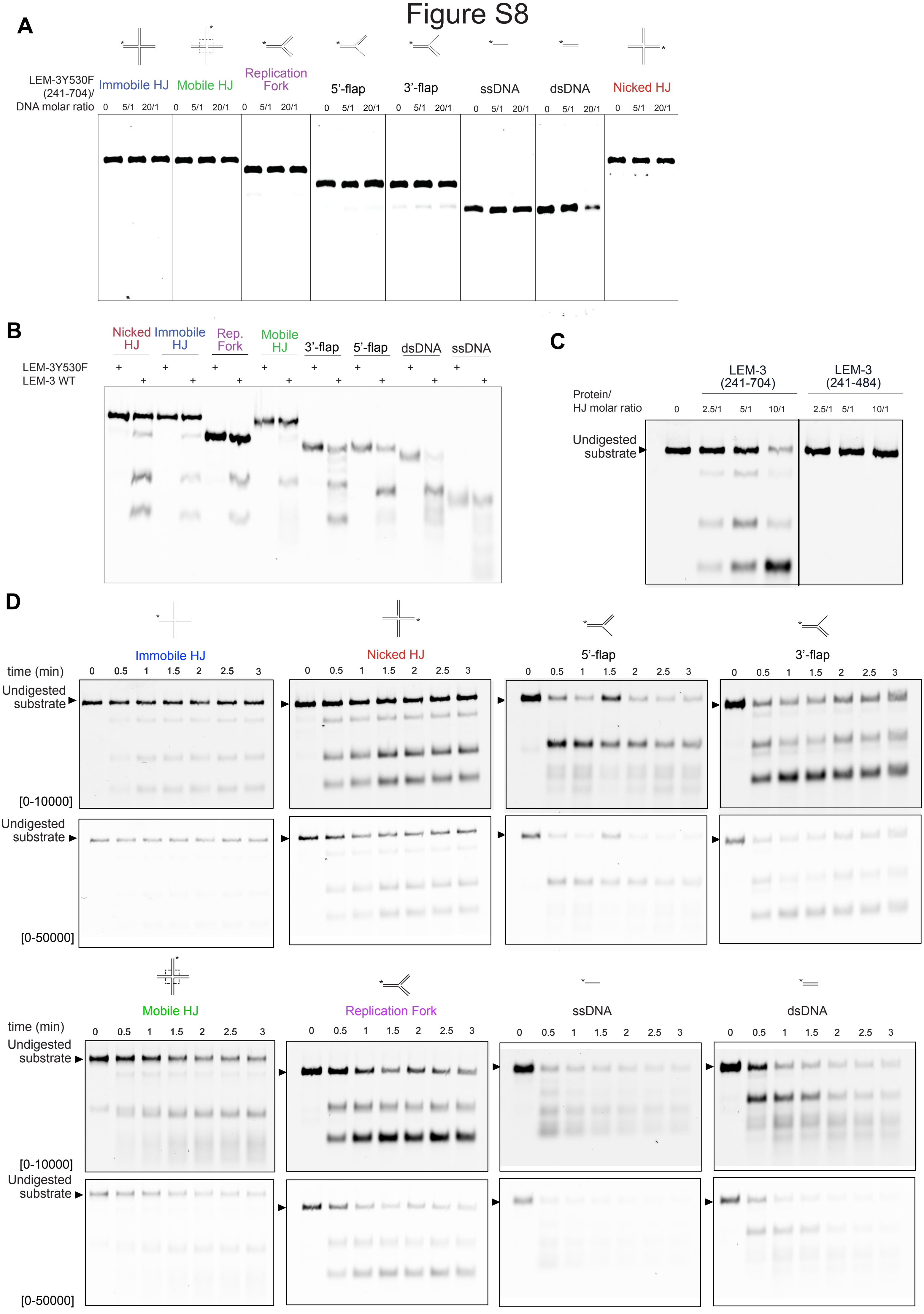
A
LEM-3Y530F (241-704)/ DNA molar ratio
Immobile HJ
Mobile HJ
Replication Fork
5'-flap
3'-flap
ssDNA
dsDNA
Nicked HJ
0 5/1 20/1
B
Nicked HJ
Immobile HJ
Rep. Fork
Mobile HJ
3'-flap
5'-flap
dsDNA
ssDNA
LEM-3Y530F
LEM-3 WT
C
LEM-3 (241-704)
LEM-3 (241-484)
Protein/ HJ molar ratio
0
2.5/1
5/1
10/1
Undigested substrate
D
Immobile HJ
Nicked HJ
5'-flap
3'-flap
time (min)
0 0.5 1 1.5 2 2.5 3
Undigested substrate
[0-10000]
[0-50000]
Mobile HJ
Replication Fork
ssDNA
dsDNA

Figure S8: Analysis of the specificity of the LEM-3 nuclease (related to Figure 3). (A-B) Nuclease activity of LEM-3Y530F(241-704) on various model DNA substrates. The purified protein used for this experiment is indicated in Figure S1B (for panel A) and S1F (for panel B) (n=1 experiment each). (C) Nuclease activity of LEM-3(241-704) and LEM-3(241-484) on HJ substrates. The purified protein used for this experiment is indicated in Figure S1E,G (n=1 experiment) **(D)** Kinetic experiment of LEM-3 with different DNA substrates. The purified protein used for this experiment is indicated in Figure S1E. LEM-3(241-704) was incubated with the different DNA substrates at a molar ratio of 5:1 for 0 to 3 min. The cleavage products were analyzed using a 6% neutral PAGE. The size of the undigested DNA substrates is indicated. (1 representative experiment is shown for each substrate, in total n=3 independent experiments).

Figure S9

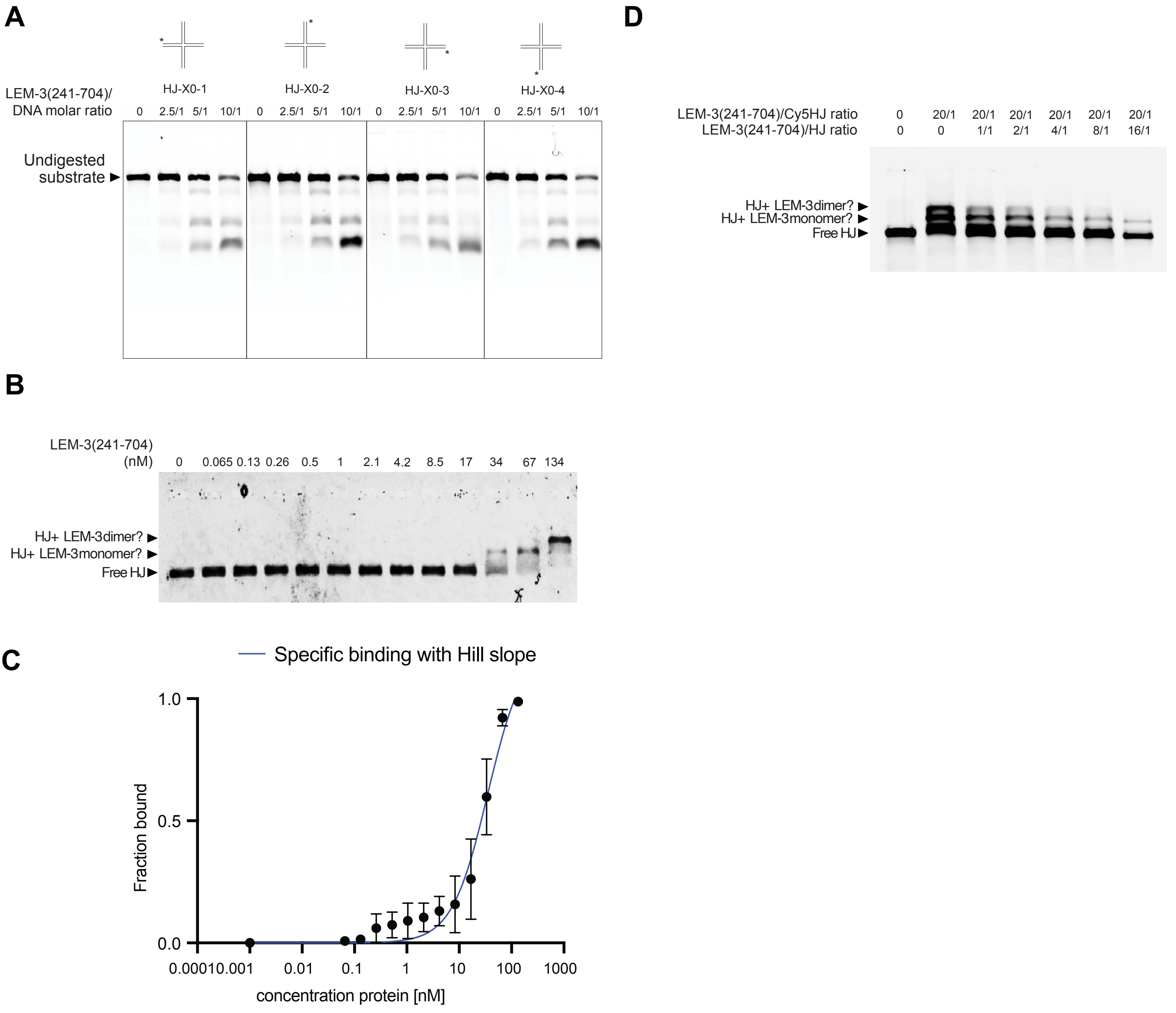

A
HJ-X0-1
HJ-X0-2
HJ-X0-3
HJ-X0-4
LEM-3(241-704)/
DNA molar ratio
0 2.5/1 5/1 10/1 0 2.5/1 5/1 10/1 0 2.5/1 5/1 10/1 0 2.5/1 5/1 10/1
Undigested substrate
B
LEM-3(241-704) (nM)
0 0.065 0.13 0.26 0.5 1 2.1 4.2 8.5 17 34 67 134
HJ+ LEM-3dimer?
HJ+ LEM-3monomer?
Free HJ
C
Specific binding with Hill slope
Fraction bound
0.0
0.5
1.0
0.00010.001 0.01 0.1 1 10 100 1000
concentration protein [nM]
D
LEM-3(241-704)/Cy5HJ ratio
0 20/1 20/1 20/1 20/1 20/1 20/1
LEM-3(241-704)/HJ ratio
0 0 1/1 2/1 4/1 8/1 16/1
HJ+ LEM-3dimer?
HJ+ LEM-3monomer?
Free HJ

Figure S9: Mapping of the cleavage site of HJ by LEM-3 and measuring the affinity of LEM-3 for HJ (related to Figure 3). (A) Nuclease activity of LEM-3(241-704) on HJ, where the labeling was performed on each of the 4 strands. The purified protein used for this experiment is indicated in Figure S1A. Different protein/DNA substrate molar ratios were used, and incubation was performed at 37 C for 10 min. The cleavage products were analyzed using a 6% neutral PAGE. The size of the undigested DNA substrates is indicated. (representative images are shown, n=4 experiments). **(B)** Various concentrations of LEM-3 protein were incubated with 0.13nM HJ-X0-1, and the resulting products were analyzed on a 6% neutral PAGE. The purified protein used for this experiment is indicated in Figure S1A. (representative images are shown, n=3 experiments). **(C)** The HJ bound fraction was plotted as a function of LEM-3(241-704) concentration, and the data was fitted to the Hill equation**. (D)** Labeled HJ-X0-1 (Cy5HJ) was incubated with LEM-3(241-704), and the ratio of shifted protein was measured by gel shift assay in the presence of increasing amounts of unlabeled HJ substrate. The purified protein used for this experiment is indicated in Figure S1A. (n=1 experiment).

Figure S10

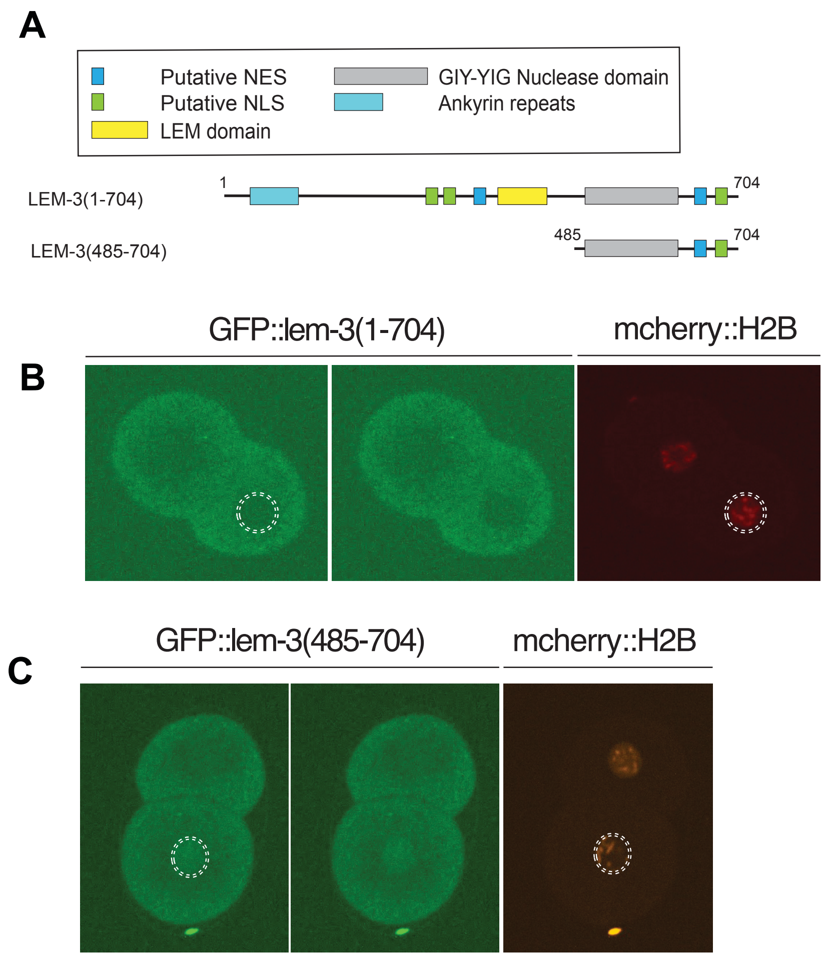
A
Putative NES
Putative NLS
LEM domain
GIY-YIG Nuclease domain
Ankyrin repeats
LEM-3(1-704)
1
704
LEM-3(485-704)
485
704
B
GFP::lem-3(1-704)
mcherry::H2B
C
GFP::lem-3(485-704)
mcherry::H2B

Figure S10: LEM-3(485-704) derivative partially mis-localized to the nucleus. (A) Schematic of the different LEM-3 derivatives used in this figure. **(B)** GFP::LEM-3(1-704) is excluded from the nucleus (dotted circle). **(C)** GFP:LEM-3 (485-704) is not excluded from the nucleus (dotted circle).

Figure S11

A Alignment of LEM domain in ANKLE1 family

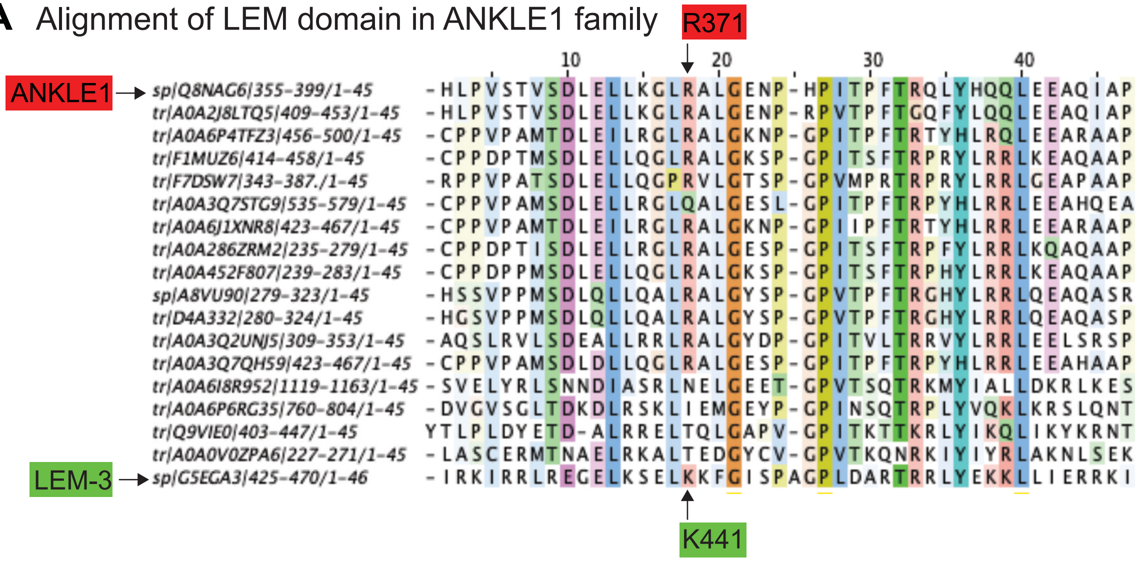

B Alignment of LEM domain in ANKLE1 family to LEM like domain in LAP2 family

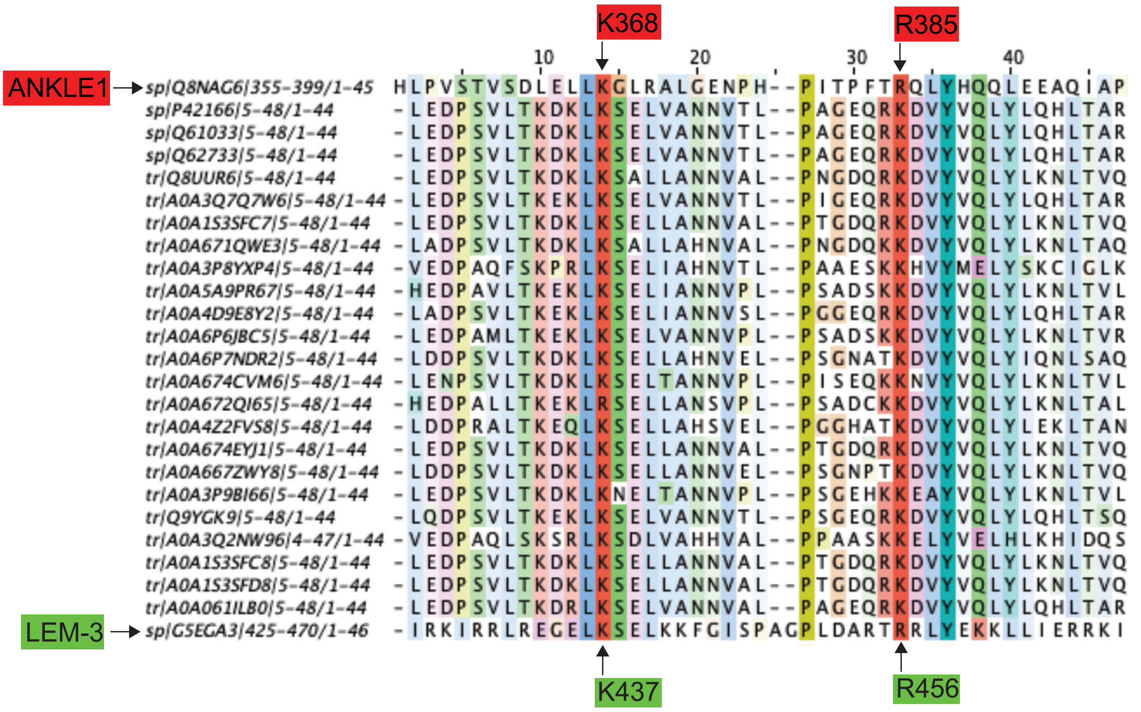

C Alignment of LEM domain in ANKLE1 family to LEM domain in LAP2 family

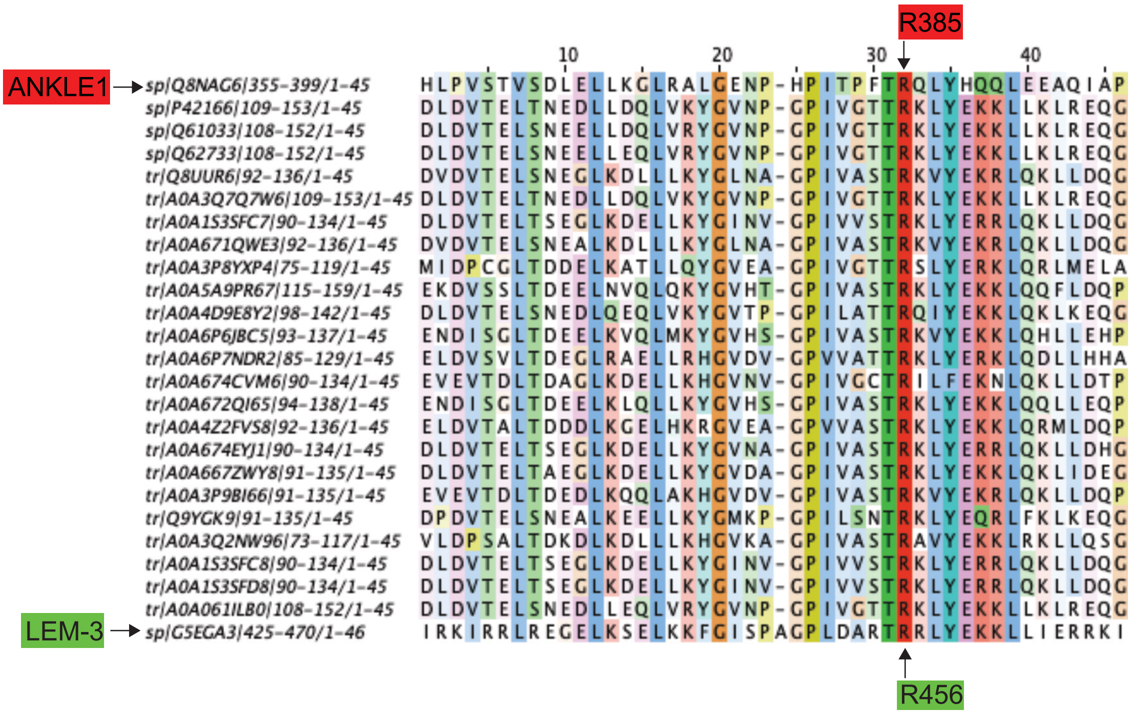

Figure S11: Sequence alignments of LEM and LEM-like domains. (A) Alignment of LEM domain in ANKLE1 family. **(B)** Alignment of LEM domain in ANKLE1 family to LEM-like domain in LAP2 family. **(C)** Alignment of LEM domain in ANKLE1 family to LEM domain in LAP2 family.

Figure S12

A

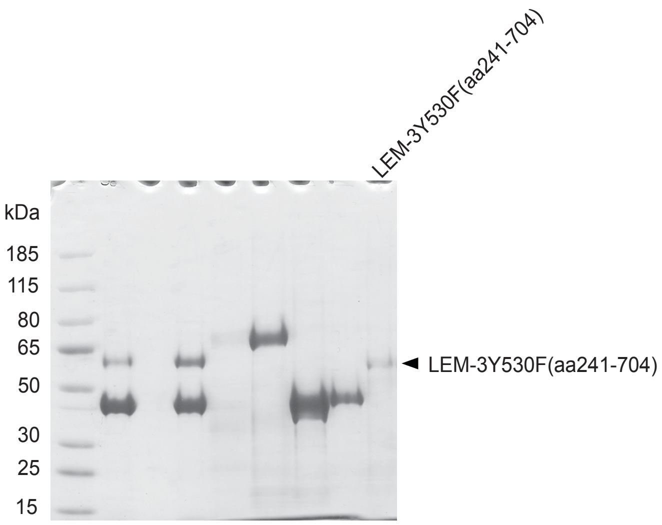

B

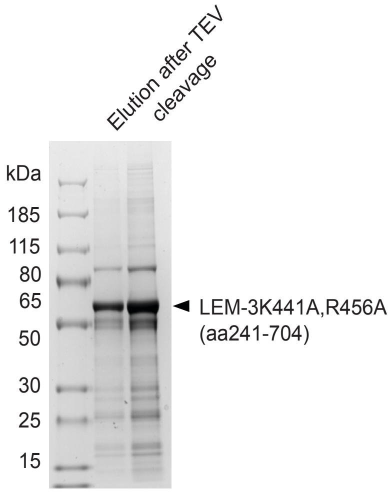

C

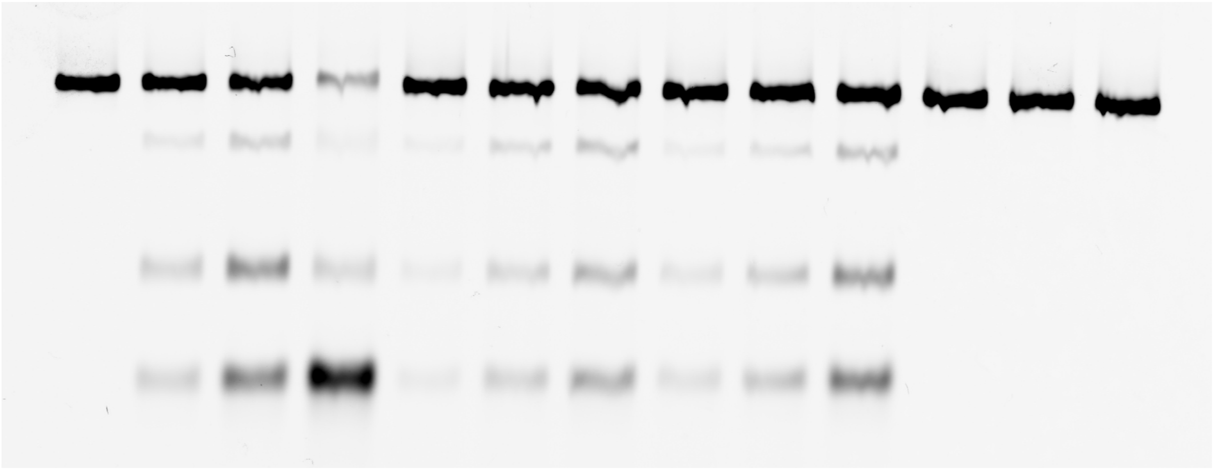

Figure S12: Uncropped image of coomassie gel. (A) uncropped image of Figure S1B **(B)** uncropped image of Figure S1D. **(C)** uncropped image of Figure S8C.

Table S1: List of strains used in this study

Strain name	Genotype	Source
N2	Wild-type	CGC
TG4397	*lem-3(cop859 [Plem-3:GFP::Stag::lem-3(1-704):3'UTRlem-3]) I ; odIs57 [Ppie-1:mcherry::H2B]*	Gartner lab
SP483	*lem-3 (mn155) I (premature stop codon at R190)*	CGC
TG4318	*lem-3(op444) I*	Ye Hong
PHX1970	*lem-3(syb1970 [Plem-3:GFP::Stag::lem-3E620A(1-704):3'UTRlem-3]) I ; odIs57 [Ppie-1:mcherry::H2B].*<br>*Generated in the background of TG4397 lem-3(cop859).*	SunyBiotech
PHX2062	*lem-3(syb2062 [Plem-3:GFP::Stag::lem-3Y556F(1-704):3'UTRlem-3]) I ; odIs57 [Ppie-1:mcherry::H2B].*<br>*Generated in the background of TG4397 lem-3(cop859).*	SunyBiotech
PHX1949	*lem-3(syb1949 [Plem-3:GFP::Stag::lem-3Y530F(1-704):3'UTRlem-3]) I ; odIs57 [Ppie-1:mcherry::H2B].*<br>*Generated in the background of TG4397 lem-3(cop859).*	SunyBiotech
PHX4324	*lem-3(syb4324 [Plem-3:GFP::Stag::lem-3(1-524,637-704):3'UTRlem-3]) I ; odIs57 [Ppie-1:mcherry::H2B].*<br>*Generated in the background of TG4397 lem-3(cop859).*	SunyBiotech
PHX3292	*lem-3(syb3292 [Plem-3:GFP::Stag::lem-3(485-704):3'UTRlem-3]) I ; odIs57 [Ppie-1:mcherry::H2B].*<br>*Generated in the background of TG4397 lem-3(cop859).*	SunyBiotech
PHX1935	*lem-3(syb1935 [Plem-3:GFP::Stag::lem-3(1-424,471-704):3'UTRlem-3]) I ; odIs57 [Ppie-1:mcherry::H2B].*<br>*Generated in the background of TG4397 lem-3(cop859).*	SunyBiotech
PHX2728	*lem-3(syb2728 [Plem-3:GFP::Stag::lem-3(241-704):3'UTRlem-3]) I ; odIs57 [Ppie-1:mcherry::H2B].*<br>*Generated in the background of TG4397 lem-3(cop859).*	SunyBiotech
PHX7187	*lem-3(syb7187[Plem-3::GFP::Stag::lem-3Y530F(485-704):3'UTRlem-3]) I ; odIs57 [Ppie-1:mcherry::H2B].*<br>*Generated in the background of TG4397 lem-3(syb3292).*	SunyBiotech
PHX6759	*lem-3(syb6759{full KO]) I.*<br>*Generated in the background of N2(wt).*	SunyBiotech
TG4884	*lem-3(gt3440[Plem-3::GFP::Stag::lem-3Y530F(241-704):3'UTRlem- 3]) I ; odIs57 [Ppie-1:mcherry::H2B].*<br>*Generated in the background of TG4397 lem-3(syb2728).*	Stéphane Rolland
TG4887	*lem-3(gt3443[Plem-3::GFP::Stag::lem-3K441A;R456A(241-704):3'UTRlem-3]) I ;odIs57[Ppie1:mcherry::H2B].*	Stéphane Rolland

	Generated in the background of TG4861 lem-3(gt3420).	
TG4892	*lem-3(gt3445[Plem-3::GFP::Stag::lem-3(241-424,471-704):3'UTRlem-3])I ;odIs57[Ppie1:mcherry::H2B].*<br>*Generated in the background of TG4397 lem-3(syb2728).*	Stéphane Rolland
PHX7665	*lem-3(syb7665[Plem-3::GFP::Stag::lem-3(1-25,139-704):3'UTRlem-3]) I ; odIs57 [Ppie-1:mcherry::H2B].*<br>*Generated in the background of TG4397 lem-3(cop859).*	SunyBiotech
TG4688	*lem-3(gt3382[Plem-3::eGFP::Stag::lem-3(K441A;R456A)]) I; odIs57 [Ppie-1:mcherry::H2B].*<br>*Generated in the background of TG4397 lem-3(cop859).*	Stéphane Rolland
PHX7973	*lem-3(syb7973[Plem-3::GFP::Stag::lem-3(1-424):3'UTRlem-3]) I ; odIs57 [Ppie-1:mcherry::H2B].*<br>*Generated in the background of TG4397 lem-3(cop859).*	SunyBiotech
TG4584	*lem-3(gt3329[Plem-3::GFP::Stag::lem-3(1-704)::3xHA:3'UTRlem-3]) I ; odIs57 [Ppie-1:mcherry::H2B].*<br>*Generated in the background of TG4397 lem-3(cop859).*	Stéphane Rolland
TG4894	*lem-3(gt3447[Plem-3::GFP::Stag::lem-3(241-704)::3xHA:3'UTRlem-3]) I ; odIs57 [Ppie-1:mcherry::H2B].*<br>*Generated in the background of PHX2728 lem-3(syb2728).*	Stéphane Rolland
TG4969	*lem-3(gt3500[Plem-3::GFP::Stag::lem-3Y530F(241-704)::3xHA:3'UTRlem-3]) I ; odIs57 [Ppie-1:mcherry::H2B].*<br>*Generated in the background of TG4884 lem-3(gt3440).*	Stéphane Rolland
TG4968	*lem-3(gt3499[Plem-3::GFP::Stag::lem-3(1-25,139-704)::3xHA:3'UTRlem-3]) I ; odIs57 [Ppie-1:mcherry::H2B].*<br>*Generated in the background of PHX7665 lem-3(syb7665).*	Stéphane Rolland
TG4828	*lem-3(gt3403[Plem-3::GFP::Stag::lem-3Y530F(1-704)::3xHA:3'UTRlem-3]) I ; odIs57 [Ppie-1:mcherry::H2B].*<br>*Generated in the background of PHX1949 lem-3(syb1949)*	Stéphane Rolland
TG4896	*lem-3(gt3449 [Plem-3:GFP::Stag::lem-3(1-524,637-704)::3xHA:3'UTRlem- 3]) I ; odIs57 [Ppie-1:mcherry::H2B].*<br>*Generated in the background of PHX4324 lem-3(syb4324).*	Stéphane Rolland
TG4897	*lem-3(gt3450 [Plem-3:GFP::Stag::lem-3(485-704)::3xHA:3'UTRlem- 3]) I ; odIs57 [Ppie-1:mcherry::H2B].*<br>*Generated in the background of PHX3292 lem-3(syb3292).*	Stéphane Rolland
TG4951	*lem-3(gt3489[Plem-3::GFP::Stag::lem-3(241-424,471-704)::3xHA:3'UTRlem- 3]) I ;odIs57[Ppie1:mcherry::H2B].*<br>*Generated in the background of TG4892 lem-3(gt3445).*	Stéphane Rolland
TG4952	*lem-3(gt3491[Plem-3::GFP::Stag::lem-3K441A;R456A(241-704)::3xHA :3'UTRlem- 3]) I ;odIs57[Ppie1:mcherry::H2B].*<br>*Generated in the background of TG4887 lem-3(gt3443).*	Stéphane Rolland
TG4965	*lem-3(gt3497 [Plem-3:GFP::Stag::lem-3(L659F)::3xHA:3'UTRlem-3]) I ; odIs57 [Ppie-1:mcherry::H2B].*	Stéphane Rolland

	Generated in the background of TG4553 lem-3(gt3326)	
TG4639	*lem-3(gt3364[Plem-3::eGFP::Stag::lem-3(deltaLEM)::3xHA]) I ; odIs57 [Ppie-1:mcherry::H2B].*<br>*Generated in the background of PHX1935 lem-3(syb1935)*	Stéphane Rolland
TG4711	*lem-3(gt3396[Plem-3::eGFP::lem-3(K441A;R456A)::3HA]) I ; odIs57 [Ppie-1:mcherry::H2B].*<br>*Generated in the background of TG4688 lem-3(gt3382)*	Stéphane Rolland

Table S2. crRNA and ssODN used for genome editing

	name	sequence	Used to generate the following strains
crRNA	crRNAlem-3op444	ATGGAACATTTTTGCTGGAT**AGG**	TG4553
	crRNAlem-3-Y530F	TCGAATTTTGGGAAGCAATG**TGG**	TG4884
	crRNAlem-3-2	CTTCTTGTACGAGCATCCAG**AGG**	TG4861, TG4887
	crRNAlem-3-1	AAGAGCGAGCTGAAAAAGTT**CGG**	TG4887
	crRNAlem-3L	ACACCGACCACAGTCGATGA**TGG**	TG4892
	crRNAlem-3R	TACGAACCGTGGATATTCAC**CGG**	TG4892
	cRNA-lem3Cterm	GACGTGGAGCAGGTGGTGGA**CGG**	TG4584, TG4894, TG4969, TG4968, TG4828, TG4896, TG4897, TG4951, TG4953, TG4965
ssODN	ssODNlem-3op444	GCTGGGACAATATCACTAAATCAGAATATGGAAC ATTTTTTCTCGACAGgtgaacatctcgccattatttttcaatctaaa	TG4553
	ssODNlem-3-Y530F	GTGGAAATGGATTCGGATATAATGCGTTTTGCTTC CTCATTATGGATCCTCGAATTCTGGGAAGCAACGT GGAGAACCTTACACTTGAAACCTTTGTACGATCA	TG4884
	ssODN-R456A	AGCGAGCTGAAAAAGTTCGGAATCTCTCCAGCAG GACCTCTGGACGCTCGTACAGCTAGACTATATGA GAAGAAACTCCTGATTGAAAGACGGAAAATT	TG4861
	ssODN-lem-3-triple	GGAGAAATCAGAAAAATTCGACGT CTTCGAGAAG GAGAACTGGCTAGCGAGCTGGCTAAGTTCGGAAT CTCTCCAGCAGGACCTCTGGACGCTCGTACAGCT AGACTATATGAGAAGAAACTCCTGATTGAAAGAC GGAAAATT	TG4887
	ssODNdeltaLEM	TCATCTGCGGAAGACGACAAAGAAGCAGAAGTAT CAACACCGACCACAGTTGACGATGGAGAAACGAA CCGTGGATACTCGCCGGATGCTGACGTTGTTTCA TGTGTAAGTTATATATTTTCATTTCTC	TG4892
	ssODNlem-3HA	TTTATCCATATGTGAACAATCGACGTGGAGCAGG TGGTGGGCGGACACCAAAAACACCGAAATACCC ATACGACGTCCCAGACTACGCCTACCCATATGAT GTCCCGGATTACGCTTACCCATACGATGTTCCAG ATTACGCTTAATATAAATACTTCATTTATTCCATAT GTTTATATTTCA	TG4584, TG4894, TG4969, TG4968, TG4828, TG4896, TG4897, TG4951, TG4953, TG4965

Table S3. MultiBac expression vectors for LEM-3 production

Plasmids	MW of LEM-3 protein
pFL-His-GST-TEV-GSM-LEM3$^{1-704}$	78.2kDa
pFL-His-GST-TEV-GSM-LEM3$^{241-704}$	52kDa
pFL-His-GST-TEV-GSM-LEM3$^{241-704}$-Y530F	52kDa
pFL-His-GST-TEV-GSM-LEM-3$^{241-704}$-R456A	52kDa
pFL-His-GST-TEV-GSM-LEM-3$^{241-704}$-K441A-R456A	52kDa
pFL-His-GST-TEV-GSM-LEM3$^{341-704}$	41kDa
pFL-His-GST-TEV-GSM-LEM-3$^{485-704}$	24kDa

Table S4. Sequences of oligonucleotides for synthetic DNA substrates (Wyatt et al. 2013).

Oligo	Sequence (5'-3')
X0-1	ACGCTGCCGAATTCTACCAGTGCCTTGCTAGGACATCTTTGCCCACCTGCAGGTTCACCC
X0-2	GGGTGAACCTGCAGGTGGGCAAAGATGTCCATCTGTTGTAATCGTCAAGCTTTATGCCGT
X0-3	ACGGCATAAAGCTTGACGATTACAACAGATCATGGAGCTGTCTAGAGGATCCGACTATCG
X0-4	CGATAGTCGGATCCTCTAGACAGCTCCATGTAGCAAGGCACTGGTAGAATTCGGCAGCG T
X26-1	GCGCTACCAGTGATCACCAATGGATTGCTAGGACATCTTTGCCCACCTGCAGGTTCACCC
X26-2	GGGTGAACCTGCAGGTGGGCAAAGATGTCCTAGCAATCCATTGTCTATGACGTCAAGCTC
X26-3	GAGCTTGACGTCATAGACAATGGATTGCTAGGACATCTTTGCCGTCTTGTCAATATCGGC
X26-4	GCCGATATTGACAAGACGGCAAAGATGTCCTAGCAATCCATTGGTGATCACTGGTAGCGC
X0-2.5	GGGTGAACCTGCAGGTGGGCAAAGATGTCC
X0-3.5	CATGGAGCTGTCTAGAGGATCCGACTATCG
X0-1.32	ACGCTGCCGAATTCTACCAGTGCCTTGCTAGG
X0-1.28**	ACATCTTTGCCCACCTGCAGGTTCACCC

ssDNA= X0-1*
Duplex= X0-1* + X0-4
Immobile Holliday Junction (HJ)-HJ X0-1 = X0-1* + X0-2 + X0-3 + X0-4
Immobile HJ-HJ X0-2 = X0-1 + X0-2* + X0-3 + X0-4
Immobile HJ-HJ X0-3 = X0-1 + X0-2 + X0-3* + X0-4
Immobile HJ-HJ X0-4 = X0-1 + X0-2 + X0-3 + X0-4*
Mobile HJ-HJ X26 = X26-1* + X26-2 + X26-3 + X26-4
Nicked HJ= X0-1.32 + X0-1.28** + X0-2 + X0-3* + X0-4
Replication Fork (RF)= X0-1* + X0-2.5 + X0-3.5+ X0-4
5'-flap= X0-1*+ X0-2.5 + X0-4
3'-flap=X0-1*+ X0-3.5 + X0-4
* The oligonucleotide is labelled by 5'-Cy5
** This oligonucleotide carries a 5'-phosphate group
